# Impact of Aggregation Functions and Learning Settings in Privacy‐Preserving Federated Learning for Skin Cancer Detection

**DOI:** 10.1049/htl2.70096

**Published:** 2026-08-31

**Authors:** Bela Shrimali, Rebakah Geddam, Hemant Ghayvat, Rushi Shah, Mohit Ginglani

**Affiliations:** ^1^ Unitedworld Institute of Technology Karnavati University Gandhinagar India; ^2^ Computer Science and Media Technology Department Faculty of Technology Linnaeus University Växjö Sweden; ^3^ IMT, Department of Humanities and Technology Roskilde University Roskilde Denmark; ^4^ Institute of Technology Nirma University Gujarat India

**Keywords:** AI in healthcare, convolutional neural networks, deep learning, dermatology, image classification, ISIC dataset, medical image analysis, skin cancer detection

## Abstract

Skin cancer is among the most common malignant tumors worldwide, and early detection is essential to improve patient survival and recovery. Conventional AI‐based diagnostic approaches rely on centralized data, which raises security and privacy concerns. This paper presents an implementation of federated learning that addresses these challenges by enabling collaborative model training among simulated distributed client nodes while ensuring that raw patient data remains confidential. A convolutional neural network architecture, ResNet50, is employed at client nodes, with preprocessing steps including image augmentation, contrast enhancement, and lesion segmentation to improve feature extraction on the International Skin Imaging Collaboration (ISIC) dataset. Multiple aggregation algorithms are implemented at the central server, along with both vertical and horizontal federated learning settings. Their performance is evaluated using metrics such as precision, accuracy, recall, and F1‐score. In the horizontal federated learning setting, the FedNova aggregation approach outperformed other methods, achieving an accuracy of 76.45%, F1‐score of 0.73, and a recall of 0.72, demonstrating enhanced overall classification performance among the evaluated aggregation algorithms and stable convergence across clients. These results highlight the impact of federated learning settings and aggregation strategies on overall skin cancer detection performance.

## Graphical abstract

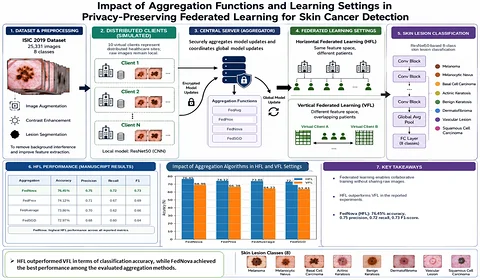

## 1 Introduction

Skin cancer is considered the most prevalent type of cancer, as reported by the International Agency for Research on Cancer (IARC). It is estimated that around 1.5 million new cases have been reported in the past years [1]. The diagnosis of skin cancer involves several stages, such as a consultation with a specialist, dermoscopy, close examination of tissue under a microscope, genetic information gathering, and, in difficult cases, a radiological exam. Each stage is extremely important to make a correct diagnosis and for the selection of proper treatment. The first diagnostic stage involves a visit to a dermatologist who inspects the skin for any abnormal growths. They will analyze and observe the skin, looking for features like its composition—the presence of asymmetry, different colors, outline of a mole, its size, and any changes in the dynamics of the mole over time. Such characteristics will help the doctor distinguish harmful and non‐harmful skin lumps. There are further signs that would indicate that the mole could be malignant, like bleeding, open wounds, rapid changes in shape, the patient's medical history, etc.

Dermatoscopy helps to visualize the skin layers, which cannot be seen by the naked eye. A dermatoscope is used to identify skin patterns of possible cancers. It enhances diagnostic precision, thus preventing the need for unnecessary biopsy. In advanced cases, molecular tests are performed to detect specific gene mutations, thus allowing targeted therapy. Imaging studies such as ultrasound, magnetic resonance imaging, computed tomography, and positron emission tomography scans are used as well to determine whether cancer has spread to nearby organs.

Although technological enhancement, traditional screening methods remain with many concerns. In developing countries, people are often unable to find dermatology specialists who have the necessary diagnostic equipment, especially in remote areas. Human interpretation in diagnosis may be prone to misdiagnosis. Deep learning techniques/ models, a core part of a subset of artificial intelligence (AI), resolve these and similar problems. These models can automatically process numerous features from clinical and dermatoscopic images, leading to faster and more accurate results compared to traditional methods. Nevertheless, successful application of such models requires a great variety of data, which is difficult to obtain while complying with the legislation on patient confidentiality. Federated learning helps with these privacy concerns by allowing different medical centers to train models together without sharing the actual patient data. Instead of sharing the raw images, only the improvements in the model are shared, which keeps the data private. This method also makes the models stronger and allows them to be used in different parts of the country. A detailed comparison of centralized learning and federated learning in healthcare is shown in Table 1.

**TABLE 1:** Comparisons: Centralized machine learning and federated learning in healthcare.

Aspect	Centralized ML	Federated learning
Data sharing	Required	Not required
Privacy risk	High	Low
Regulatory compliance	Difficult	Easier
Deployment cost	Lower	Higher
Scalability	Limited	High

Implementing federated learning in analyzing medical images has been limited either to singularly applied federated learning types or to a single aggregation technique for diagnostic purposes. To the best of our knowledge, there is a void in systematic comparisons between horizontal federated learning (HFL) and vertical federated learning (VFL) with respect to both IID and non‐IID data distributions, in addition to the multitude of aggregation algorithms available that can provide multiclass skin cancer classifications. Less complete investigations have been performed as to how the use of different federation aggregation approaches can affect classification accuracy due to a lack of harmonized experimental designs in terms of preprocessing methods, model architectures, and evaluation protocols; thus, this research will offer a controlled and comparative assessment of widely used approaches to federated learning across both HFL and VFL for privacy‐preserving skin classifications for various aggregation settings.

The prime contributions of the proposed framework are outlined as follows:

- A **privacy‐preserving FL architecture** enabling collaborative training of a CNN (ResNet50) across distributed dermatology datasets without centralized data pooling.
- A **multi‐stage preprocessing pipeline** integrating U‐Net‐based lesion segmentation, synthetic minority oversampling (SMOTE), and image augmentation for data preprocessing before federated model training.
- A **systematic comparative evaluation** of HFL and VFL is performed under both IID and non‐IID data distributions to investigate their appropriateness for privacy‐preserving skin lesion classification.
- A **controlled comparative analysis of horizontal and vertical FL** approaches, with implementation of FedAvg, FedProx, and VFL with Paillier encryption, quantifying performance gaps (IID: 92.1% vs non‐IID: 86.7%) and communication overhead (VFL requiring 2.3× more bandwidth).

The overall organization of the article is as follows: Section 2 provides a critical analysis of the existing literature in healthcare‐federated learning implementations. Section 3 presents the proposed work that discusses dataset preparation, methodology, and the federated learning pipeline. Quantitative results comparing centralized and federated models across multiple metrics are presented next in Section 4 having clinical validation. Conclusion with a discussion of limitations and future directions is covered in Section 5.

## 2 Related Work

FL has been explored for medical diagnosis due to its ability to support the privacy of personal data and a collaborative learning approach without the required direct data sharing among medical institutions. This is particularly significant in the healthcare domain, where patients' data privacy and sensitivity often restrict centralized AI implementations. Existing state‐of‐the‐art approaches have shown that FL is effectively implemented across different cancer types and diagnostic tasks. Pati et al. [2] proposed the FL for glioblastoma tumor segmentation using a ResUNet model trained across four clients. They worked on rare glioblastoma cases and utilized multi‐site BraTS data collected from multiple medical institutions. Their implementation results demonstrated that federated training improved tumor boundary detection, resulting in an improvement in F1‐score. The diagnosis of breast cancer has been one of the most popular areas explored within the field of federated learning. Jimenez et al. [3] proposed a CNN‐based architecture and employed memory‐aware curriculum learning techniques for mammogram classification. Their approach demonstrated improved accuracy and recall rates thanks to their iterative model updating approach. Almufareh et al. [4] proposed a federated deep learning architecture for breast cancer detection when working with the Wisconsin breast cancer diagnostic dataset. They worked with two clients and achieved decent accuracy, precision, and recall rates. There have also been studies focusing on developing generic federated learning frameworks for cancer diagnosis. Ma et al. [5] proposed an approach that utilizes federated learning and is able to work with several types of cancer. Their CNN‐based model demonstrated good diagnostic performance. AlSalman et al. [6] also evaluated a deep learning model based on a convolutional architecture and used mammography data from several different sources. Their findings showed the effectiveness of using federated learning while working with large and diverse imaging data for obtaining accurate diagnoses. An Inception‐V3 model was employed by Hossain et al. [7] in a collaborative federated environment to classify lung and colon cancer images with considerable success in the multi‐class categorization area. Notably, in another work, blockchain technology was incorporated into federated learning by Heidari et al. [8] to enhance trust and safety in lung cancer detection systems. Moreover, the use of ensemble learning in combination with federated learning has also been investigated [9, 10]. An ensemble method‐based framework was developed by Subashchandrabose et al. [11], which merges neural network classifiers with classical machine learning classifiers for the classification of lung cancer. Their approach proved the effectiveness of ensemble models in federated machine learning. As for histopathology, Peta et al. [12] created a fully automated federated CNN framework for breast cancer classification based on the BreakHis dataset, which yielded powerful outcomes on high‐resolution histopathological images. Federated learning has also been used in cervical cancer screening. Joynab et al. [13] a CNN‐based federated model for classifying PAP‐smear images. They used the FedAvg algorithm to aggregate model weights, and their findings showed that federated learning can achieve accurate classification while keeping patient data private. The above state‐of‐the‐art discusses the FL implementation, while recent advancements in skin cancer diagnosis technology have focused on using convolutional neural networks (CNNs) and vision transformer (ViT)‐based architectures together, allowing use of local feature extraction as well as global context input. The new CNN–ViT‐based model suggested in [14] has utilized the MetaFormer architecture, with focal self‐attention mechanisms to augment the feature extraction process while minimizing background noise and enhancing the quality of localization of the lesion. According to results using two datasets, HAM10000 and ISIC 2019, the model achieved classification accuracy of 95.01% and 92.54%, respectively, while being lightweight enough to be used in real‐time in clinical settings. At the same time, the hybrid approach presented in [15] utilized ConvNeXtV2 blocks alongside separable self‐attention mechanisms to enhance the quality of fine‐grained feature extraction and the model's ability to represent global contextual dependencies. Using transfer learning and data preparation methods on ISIC 2019 dataset, the model reached 93.48% accuracy at the expense of using only 21.92 million parameters, which is higher than other successful CNN and ViT technologies. The ConvNeXtV2‐based technology used in [16] employed focal self‐attention approaches and lightweight technology to address the class imbalance. The said technology reached an accuracy of 93.60%, thus outperforming traditional ResNet50 and SwinV2 models in overall classification quality. The conclusion of these papers indicates that the improved hybrid CNN‐Transformer system with novel attention mechanisms provides a better feature representation, accuracy of classification, and processing time in terms of automated diagnosis of different skin lesions.

Similar implementations are performed in other medical imaging applications as well. DiSCNet [17] proposes a lightweight directional split convolution architecture for brain tumor classification. GBA‐Net [18] suggests a gated bottleneck and attention mechanism‐driven architecture for ischemic stroke segmentation. The findings of these papers confirm that light architecture, the attention process, and efficient feature representation help enhance the diagnostic performance of various medical imaging modalities. However, these novel deep learning models achieved impressive results in skin lesion evaluation while storing and training data centrally. In contrast, the proposed work presents a privacy‐preserving FL‐based collaborative model to evaluate the performance of the different federated aggregation functions under the same preprocessing and testing conditions to achieve a correct balance between classification accuracy and privacy.

Overall, the reviewed literature suggests that federated learning is a practical and efficient method for privacy‐preserving cancer diagnosis across different clinical research. The evidence shows that FL can match the performance of traditional centralized methods, while also addressing important issues like data privacy, limited data availability, and differences in medical data across institutions. These findings highlight the strong potential of FL‐based systems for real‐world use in clinical settings.

Despite these advances, three main challenges continue to affect the use of FL systems [19, 20, 21, 22]:

- **Communication Latency**: Communication latency remains an issue. Although hierarchical structures help reduce bandwidth issues, federated models often need more rounds of communication to reach convergence compared to centralized models. For instance, in a literature on COVID‐19 detection, federated models needed about 23% more iterations to achieve similar results [22].
- **Data Heterogeneity**: Data that is not identically and independently distributed (non‐IID) can reduce model effectiveness. In the literature on urinary disease classification, label imbalance led to a 12% drop in accuracy compared to IID benchmarks.
- **Security Vulnerabilities**: Multi‐institutional FL systems are more exposed to threats like model inversion attacks, which require encryption measures. Homomorphic encryption schemes, such as the Paillier protocol, are needed to ensure data confidentiality.

Table 2 presents a comparative overview of federated learning adoptions in cancer diagnosis, highlighting model architectures, aggregation algorithms, total client numbers, and datasets used. The table demonstrates the versatility of FL in the medical field and the further essential challenges of FL implementations, viz. communication efficiency, heterogeneity, and communication channel security.

**TABLE 2:** Comparison of FL implementations for cancer diagnosis.

Ref	Disease focused	Model used	No. of clients	Aggregation method	Dataset	Key contribution	Performance
[2]	Glioblastoma	ResUNet	4	FedAvg	BraTS (49 sites, not publicly available)	Automatic tumor boundary detection for rare glioblastoma cases using FL.	23% improvement in F1‐score
[3]	Breast cancer	CNN	3	Average	FFDM	curriculum learning Memory‐aware strategy for breast cancer classification.	Precision–recall curve improved by 23%
[4]	Breast cancer	DNN	2	FedAvg	BCW Diagnostic	Collaborative FL‐based breast cancer detection framework.	Accuracy 97.54%, Precision 96.5%, Recall 98.0%
[5]	Any cancer	CNN	3	FedAvg	Cancer prediction dataset	Assisted diagnosis considering recurrence factors and heterogeneous data.	90% accuracy
[6]	Breast cancer	DCNN	2–5	Average	VINDR‐MAMMO, CMMD, INBREAST	Robust FL framework across large mammography datasets.	98.90% accuracy
[7]	Lung and colon cancer	Inception‐V3	5	FedAvg	LC25000	Multi‐class lung cancer and colon cancer classification using collaborative FL.	99.72% accuracy
[8]	Lung cancer	Deep Learning	3	FedAvg	CIA, KDSB, LUNA16	Blockchain‐integrated FL for global lung cancer detection using limited hospital data.	99.69% accuracy
[11]	Multi‐order lung cancer	NN, SVM, KNN, DNN	3	FedAvg	Kaggle Cancer Dataset	Ensemble FL models for reliable classification into normal, benign, and malignant classes.	89.63% accuracy
[12]	Breast cancer	CNN	3	Average	BreakHis	Automated federated framework for histopathological image classification.	95.68% accuracy
[13]	Cervical cancer	CNN	3	FedAvg	SIPaKMeD	Federated PAP‐smear classification aggregating locally trained models.	94.36% accuracy

Although existing state‐of‐the‐art has highlighted the implementation effectiveness of federated learning for several types of cancer diagnosis, there is currently a lack of comparative studies evaluating differences between different federated learning structures and methods of aggregation for multi‐class skin cancer classification. Most existing literature has assessed a given federated learning structure or aggregating technique; consequently, they cannot accurately demonstrate how learning paradigms and/or aggregation techniques are affected by those same learning paradigms/aggregation techniques when assessed in parallel and controlled experiments. To address these gaps, the paper presents a systematic comparative study of HFL versus VFL employing multiple aggregation algorithms within a common experimental framework where all data preparation, model structure (architecture), and evaluation parameters are identical across all experimental groups.

## 3 Proposed Approach

### 3.1 Dataset

Various datasets [23, 24, 25, 26] are available publicly for skin cancer diagnosis. Among all available datasets [23, 25], are the most popular for predictions. For the implementation of the proposed framework, International Skin Imaging Collaboration (ISIC) 2019 dataset [24] is used. It comprises 25,331 dermoscopic images spanning eight diagnostic categories such as basal cell carcinoma, melanoma, and benign lesions. The ISIC dataset is selected for this work over the HAM10000 dataset [27] due to its superior clinical utility, resolution, and metadata richness. Table 3 summarizes the key differences.

**TABLE 3:** Comparative analysis of ISIC 2019 and HAM10000 datasets.

Parameter	ISIC 2019	HAM10000
Size & diversity	25,331 images from eight classes, sourced from 20+ global clinical institutions. Includes rare lesion types (e.g., actinic keratosis).	10,015 images from seven classes, collected from a single institution. Limited representation of rare malignancies.
Image resolution	High‐resolution images (up to 6000×4000 pixels), enabling fine‐grained feature extraction.	Lower resolution (600×450 pixels), limiting scalability for modern architectures.
Metadata	Rich metadata: patient age, sex, anatomic site, and clinical diagnoses. Supports multimodal learning.	Minimal metadata (only lesion type and diagnosis).
Use case	Suitable for diagnostic‐grade AI models requiring granular clinical insights.	Better suited for preliminary classification tasks with limited computational resources.

#### 3.1.1 Client Construction Strategy

Since the ISIC 2019 dataset is a publicly accessible, centralized dataset, it does not offer a breakdown of the data by institution or hospital. To create a federated learning environment, the dataset was partitioned into 10 virtual clients that each represent separate healthcare institutions. These virtual clients were established solely for the conduct of experiments and have no relationship with actual hospitals/clinical sites; therefore, the dataset does not have any true institution‐ or patient‐based separation, either. The ISIC 2019 dataset was used for HFL with two different forms of dataset partitioning: IID and non‐IID. For the IID configuration, images were distributed evenly among the 10 clients while keeping the overall class distribution unchanged. For the non‐IID configuration, heterogeneity among clients is simulated using a Dirichlet distribution (α=0.5), causing the class distributions among clients to differ, reflecting the typical differences that occur between healthcare institutions.

#### 3.1.2 Preprocessing Pipeline

- **Class Balancing**: The dataset shows a *long‐tail distribution*, with melanoma (1128 samples) underrepresented compared to nevi (12,875). Stratified sampling is applied to ensure an 80:20 training‐testing split ratio to preserve the original label ratios.
- **Segmentation**: To remove background interference, lesion segmentations were performed using a U‐Net‐based framework [28] as a preprocessing step. This will allow us to extract features from only the lesions, isolating them from the surrounding healthy skin. The resulting segmented images will then be used as input to the federated learning framework. The primary focus of this work is to compare different approaches to federated learning for classifying skin cancer; hence, a detailed analysis of the segmentation model was beyond the scope of the proposed work.
- **Normalization**: For normalization, pixel values are scaled to [0,1] using min‐max normalization to stabilize training.
- **Data Augmentation**: To handle class imbalance and improve generalization, a transformation process using the Augmentations library is implemented [29], including the following:
  – Geometric: Horizontal/vertical flips, rotations ($\pm 30^{\circ }$), and elastic deformations are applied.
  – Photometric: Brightness adjustment (±20%), Gaussian noise (σ=0.05), and motion blur (kernel size = 7) is adjusted.

To avoid any data leakage when developing the model, the ISIC 2019 data was split into training and testing using stratified sample sizes of 80:20. The classes were distributed as their original distribution within the data. The training set was used to perform all training‐specific pre‐processing techniques, such as data augmentation and class balancing using SMOTE; however, these techniques were not performed on the test set. Deterministic pre‐processing techniques viz. lesion segmentation, image resizing, and pixel value normalization, were used to prepare the training and testing. All of these pre‐processing methods were performed on the training and test sets separately; no information from one of the sets was used in the other. This means that test set data remain unseen during model training and are only used for performance evaluation.

### 3.2 Experimental Protocol and Validation Strategy

The ISIC 2019 dataset has first been stratified into a training subset consisting of 80% and a testing subset consisting of 20% using a random seed (seed = 42) to preserve the original class distribution. The model was developed on the basis of this dataset, in which 10% of the training subset was kept aside as a validation dataset for the purpose of model selection, monitoring convergence, and applying early stopping criteria. The validation dataset was used only for the performance assessment and was not used in adjusting the model parameters. The independent test dataset was only used for performance evaluation and was not involved in the process of training, including its partitioning by clients, data augmentation, and model tuning, being used only once for performance evaluation. Stratified sampling was applied with the help of the train_test_split method of the scikit‐learn library with reference to the specifications of the diagnostic class labels to ensure identical implementation of the data partitioning during the process of the centralized learning, VFL, and HFL.

### 3.3 Methodology

The methodology of the proposed work is presented in this section. It begins with the description of the FL framework, encompassing both horizontal and vertical learning paradigms. A centralized learning baseline is subsequently introduced to enable comparative analysis. The section then presents the training protocol, aggregation strategies, and privacy‐preserving mechanisms. The performance of the proposed approach is assessed using evaluation metrics covered at the end of the section.

#### 3.3.1 Proposed Federated Learning Framework

**FIGURE 1:**
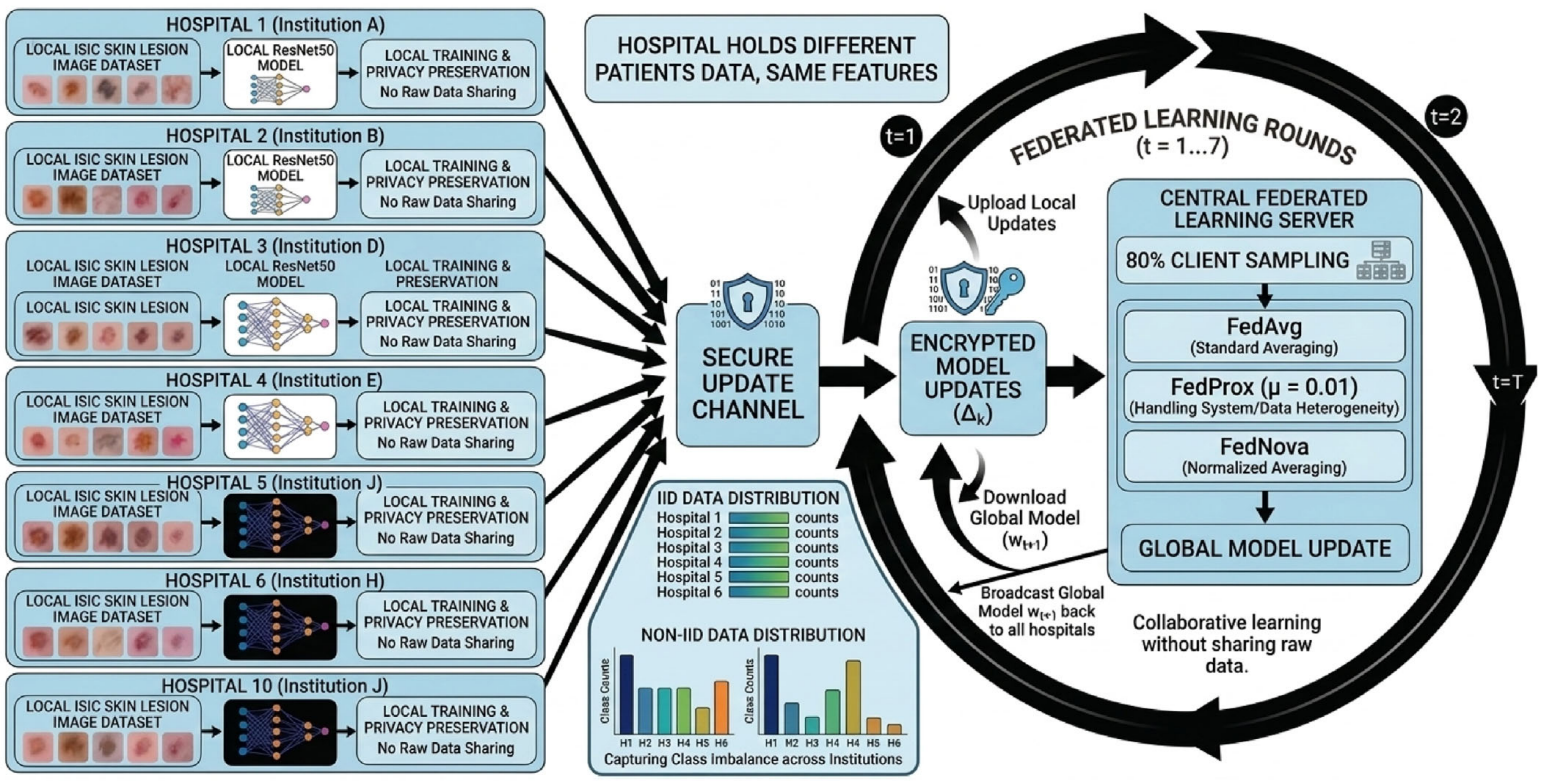
Detailed HFL workflow.

Our approach implements two variants of federated learning (horizontal and vertical) to assess the feasibility, performance, and privacy trade‐offs of skin cancer detection models trained on distributed clinical data. The description of both the variants is as follows:

- **HFL**: It is used when participating nodes/clients hold data of different patients with the same feature space. In our proposal, each hospital maintains its own collection of ISIC skin lesion images and trains a local copy of the ResNet50 model without sharing raw data. Whereas model updates are exchanged with a central server, allowing collaborative learning while preserving data ownership. To reflect realistic medical environments, IID and non‐IID data distributions are considered, with the latter capturing class imbalance commonly observed across medical institutions. The detailed HFL workflow is depicted in Figure 1.
- **VFL**: It is applied in scenarios where nodes/clients share a different feature set of overlapping patient populations. In this setting, the ResNet50 model is split between the central server and participating clients. Participating virtual clients compute feature embeddings locally and transmit only encrypted representations to the server. The remaining layers of the model are trained centrally using these embeddings, and aggregated gradients are returned to the clients. During collaboration, the coordinating server will store the “ground‐truth” labels for all clients; however, participating virtual clients will transmit encrypted intermediate feature embeddings—and not the dermoscopic images—to the coordinating server for further processing. This enables collaborative training while preventing direct access to sensitive features and labels, making it appropriate for sensitive healthcare data.
  Since the ISIC 2019 dataset is a single‐modality dermoscopy image and has not been naturally vertically partitioned with multimodal data from multiple healthcare institutions, we, therefore, implemented a simulation‐based experiment controlled using VFL. In the simulated configuration, every participating virtual client would have patients with records for the same dermoscopic lesion samples, guaranteeing full sampling overlap between all clients, thus allowing for an analytic assessment of vertically partitioned federated learning in a research environment using controlled experimental conditions despite the absence of availability of naturally vertically partitioned medical datasets.

**FIGURE 2:**
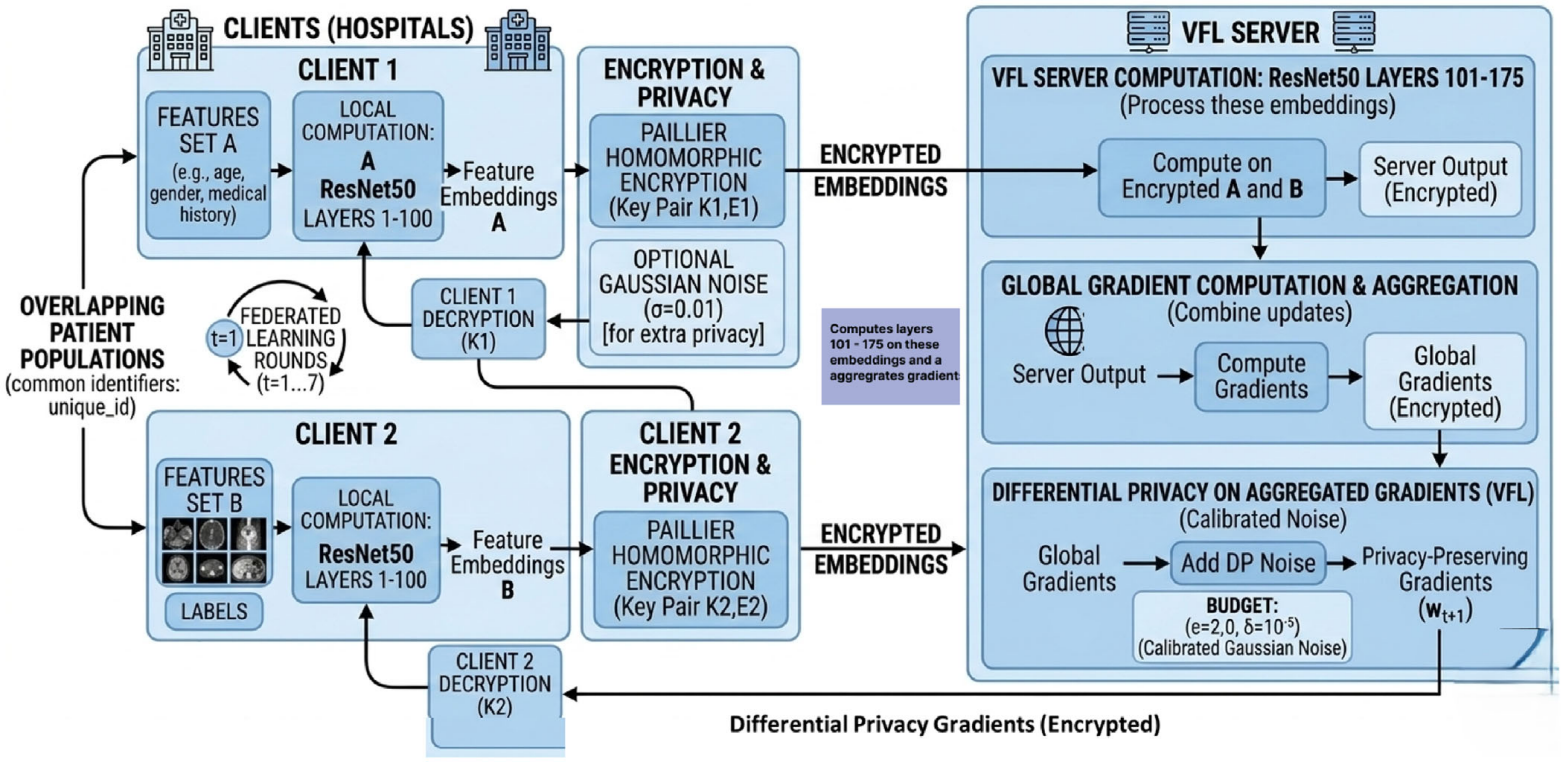
Detailed VFL workflow.

To create a realistic federated health care setting, ten different virtual federation clients were created by partitioning the ISIC 2019 dataset. Each of these clients will stand in for a simulated health care institution and only holds its own local subset of dermoscopic images. These clients are designed only for experimental purposes and do not represent real hospitals or institution specific datasets. The development of the federated learning implementation was carried out with PySyft [30] and TensorFlow Federated [31]. Every client keeps its own ISIC skin lesion images and trains a local ResNet50 copy. Upon local training, clients upload encrypted updates to a central server for aggregation without ever disclosing raw data. Detailed VFL workflow is depicted in Figure 2.

In the HFL setting, data are divided into IID (uniform splits) and non‐IID (Dirichlet distribution with α=0.5 to represent class imbalances). In every round, 80% of clients are selected randomly to join, simulating a real‐world environment. There are three aggregation algorithms—FedAvg [32], which uses client sample size weighted updates; FedProx [33], which incorporates a proximal regularizer $\frac{\mu }{2}{∥w-w_{t}∥}^{2}$ with μ=0.01; and FedNova [34], which updates by local training effort are implemented at global server.

With the simulated VFL deployment of model partitioning achieved via model partitioning instead of data partitioning, all clients share the same image samples of dermatoscopic lesions; thus, the client‐side portion of the two‐part split ResNet50 model creates intermediate feature embeddings from each image, while the server‐side portion of the two‐part ResNet50 model uses the intermediate feature embedding for the rest of the classification process. This method allowed multiple clients to collaborate via shared features, while no raw image data had to be shared.

In the VFL setup, we split ResNet50 at layer 100: clients compute feature embeddings locally and encrypt them using Paillier homomorphic encryption [35], with the option to add Gaussian noise (σ=0.01) for extra privacy. The central server computes layers 101–175 on these embeddings and sends back aggregated gradients. We again impose differential privacy by adding calibrated noise to gradients with budget ($\varepsilon =2.0,\delta =10^{-5}$). The VFL training system begins when a participating virtual client processes their local dermoscopic images through the client‐side portion of the split ResNet50 model to produce intermediate feature embeddings. Each of these intermediate feature embeddings will be encrypted using the Paillier homomorphic scheme before being sent to the coordinating server. The coordinating server will run forward propagation through the remaining layers of the network, compute the prediction loss using the centralized diagnostic label data, and compute the gradients. The coordinating server will send the encrypted gradients back to participating virtual clients to update the model parameters using their local copy of the model. It continues the process until the model convergence is achieved, allowing for the collaborative training of the model without disclosing the original dermoscopic images or the intermediate feature representations. The experimental configuration of horizontal and vertical federated implementation is summarized in Table 4.

**TABLE 4:** Experimental configuration for horizontal and vertical federated learning.

Parameter	HFL setting	VFL Setting
Clients	10 simulated federated clients	10 simulated federated clients
Data distribution	IID (uniform split) and non‐IID (Dirichlet, α=0.5)	Feature‐wise split at layer 100
Model architecture	ResNet50 (local copies at each client)	ResNet50 split: layers 1–100 (clients), 101–175 (server)
Client participation	80% of clients randomly selected per round	All clients participate
Aggregation algorithms	FedAvg, FedProx (μ=0.01), FedNova	Encrypted gradient aggregation
Privacy mechanism	Secure aggregation of model updates	Paillier homomorphic encryption
Differential privacy	Not applied	Gaussian noise (σ=0.01), $\left(\varepsilon =2.0,\delta =10^{-5}\right)$
Training process	Local training with encrypted weight updates	Clients send encrypted embeddings; server computes gradients

**FIGURE 3:**
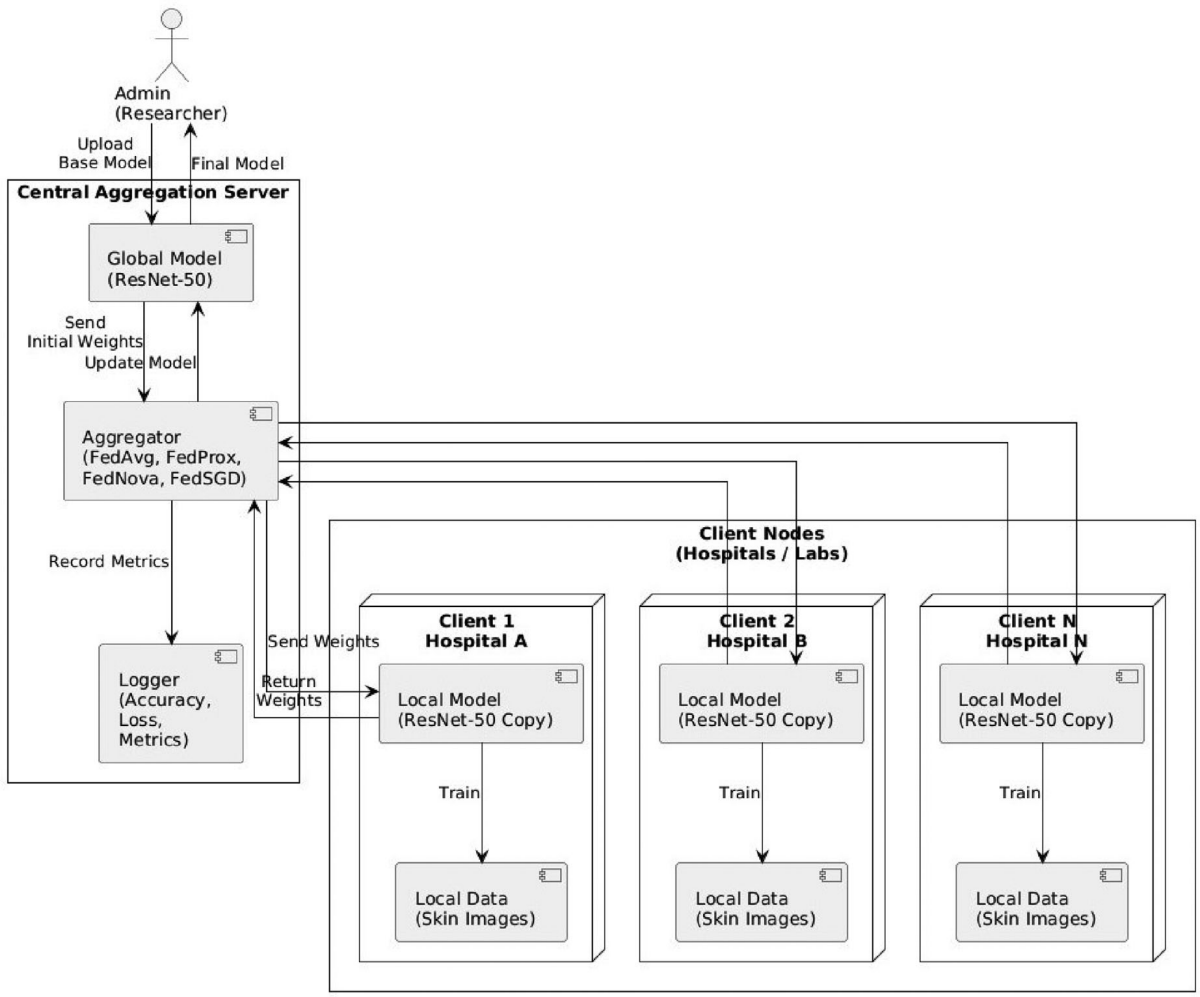
Federated learning system architecture. (A) Centralized baseline with ResNet50 backbone. (B) HFL with secure weight aggregation. (C) VFL with encrypted feature sharing. Dashed arrows denote privacy‐preserving channels.

Figure 3 illustrates the overall system architecture: at its center is a standardized convolutional backbone (ResNet50), which in the centralized case is trained on aggregated data, and in the federated cases is divided or combined across institutions under strict privacy constraints.

#### 3.3.2 Experimental Rigor and Statistical Analysis

To standardize the training pipeline and set fixed random seeds for all parameter settings in the experiments, every experimental setup (centralized learning, HFL, and VFL with different aggregation methods) was run multiple times with different random seeds (42, 52, 62, 72, and 82). The classification results are presented as averages because all the obtained results were averaged over several experimental runs. To quantify the experimental variability, standard deviation and 95% confidence interval were computed under Student's t‐distribution.

#### 3.3.3 Centralized Learning Baseline

We start with a centralized benchmark by employing a ResNet50 model that has been pretrained on ImageNet‐21k. We replace the final pooling and classification layers with a global average pooling that reduces the 2048‐dimensional feature map to 512 dimensions, and a fully connected layer mapping to eight diagnostic classes and a softmax activation. Training is performed in two stages: during the first stage, the initial 120 layers are frozen while the new head is trained for 20 epochs with a learning rate of $1\times 10^{-3}$; during the second stage, all the layers are unfrozen and fine‐tuned for 40 extra epochs with cosine annealing from $1\times 10^{-3}$ to $1\times 10^{-5}$. To prevent overfitting, we apply L2 weight decay (λ=0.001) to the classification head, spatial dropout (rate 0.5) after the final convolutional block, and label smoothing (ε=0.1). Optimizer is conducted using AdamW [36] ($\beta_{1}=0.9$, $\beta_{2}=0.999$, weight decay 0.01) in a batch size of 32 in mixed‐precision training.

#### 3.3.4 Training Protocol

All federated experiments are run for 50 communication rounds. Active clients run five local epochs per round with SGD (learning rate = 0.01) and gradient clipping of ${∥\alpha ∥}_{2}\leq 1.0$. Encrypted updates (model weights in HFL, embeddings in VFL) are sent over a network simulated by NS‐3 (latency from a Gamma distribution, mean = 125 ms; packet loss 3%), aggregated on the server and broadcast back to clients. This asynchronous configuration supports up to three rounds of staleness of updates. Table 5 summarizes all the experimental hyperparameters and federated learning configurations, and Table 6 presents the experimental configuration of computational and communication characteristics of the proposed FL framework. As presented in the table, each of the clients involved in the process sends about 102.4 MB of the model's parameters in every communication round. Hence, the total communication volume per round is around 819.2 MB with eight clients involved and approximately 40.96 GB throughout the 50 rounds. Secure aggregation was incorporated in the HFL scenario, and the VFL technique employed Paillier homomorphic encryption and differential privacy mechanisms for enhancing privacy against data leakage or unauthorized access. All the experiments were conducted in a simulated federated environment space; the latency caused by encryption was not examined.

**TABLE 5:** Experimental configuration for federated learning experiments.

Parameters used	Values
Model architecture	ResNet50
Federated learning variants	Horizontal FL (HFL), vertical FL (VFL)
Number of clients	10 simulated federated clients
Client participation (HFL)	80% of clients per round
Client participation (VFL)	All clients participate
Data distribution (HFL)	IID and non‐IID (Dirichlet, α=0.5)
Model split (VFL)	Layers 1–100 (clients), 101–175 (server)
Local training epochs	5 epochs per communication round
Total communication rounds	50
Optimizer (federated training)	SGD
Learning rate	0.01
Gradient clipping	${∥\alpha ∥}_{2}\leq 1.0$
Aggregation algorithms	FedAvg, FedProx (μ=0.01), FedNova, FedSGD
Privacy mechanism (VFL)	Paillier homomorphic encryption
Differential privacy (VFL)	Gaussian noise (σ=0.01), $\left(\varepsilon =2.0,\delta =10^{-5}\right)$
Network simulation	NS‐3 (mean latency 125 ms, packet loss 3%)

**TABLE 6:** Experimental configuration for computational and communication characteristics of the proposed FL framework.

Metric	Value / Description
Backbone network	ResNet50
Input image size	224×224×3
Total model parameters	Approximately 25.6 million
Trainable parameters	16,392 (only the final fully connected layer is fine‐tuned)
Computational complexity	Approximately 4.1 GFLOPs per inference
Number of simulated clients	10
Client participation rate	80% (8 clients per communication round)
Communication rounds	50
Estimated communication cost per client per round	Approximately 102.4 MB (assuming 32‐bit floating‐point model parameters)
Estimated communication cost per round	Approximately 819.2 MB (8 participating clients)
Estimated total communication (50 rounds)	Approximately 40.96 GB
Privacy mechanisms	Secure aggregation (HFL); Paillier Homomorphic Encryption and Differential Privacy (VFL)
Client‐side memory usage	Not separately profiled (simulated federated environment)
Communication latency	Not experimentally measured
Encryption overhead	Paillier Homomorphic Encryption implemented; computational overhead not separately profiled
Privacy–utility trade‐off	Raw patient images remain at participating institutions while achieving competitive classification performance through collaborative federated learning.

**Experimental Rigor and Statistical Analysis**: To enhance reproducibility and reliability in the experimental evaluation, a reproducible training pipeline that employs the use of random seeds has been utilized; the centralised ResNet50 was tested independently three times, and results were averaged. The same dataset partitioning and data processing procedures were used for each test. The results are given in terms of average accuracy and standard deviation of the results.

#### 3.3.5 Aggregation Algorithms

In the federated learning approach, model updates are generated independently by multiple clients/nodes and are combined at a central server using an aggregation algorithm. The selection of an aggregation algorithm is critical for determining convergence behavior and overall model performance, particularly in heterogeneous data settings common in medical applications. The description of these algorithms is as follows:

- **Federated Averaging (FedAvg)**: Federated Averaging aggregates local trained models by calculating a weighted average depending on the number of samples at each client:
(1) $$w_{t+1}=\sum_{k=1}^{K}\frac{n_{k}}{n}w_{t}^{\left(k\right)}$$
  where $w_{t}^{\left(k\right)}$ are the local model parameters obtained after training at client k. FedAvg is identified as the baseline aggregation method in federated learning [32].
- **Federated Proximal (FedProx)**: By including a proximal term in the local objective function, FedProx expands FedAvg to alleviate the effects of data heterogeneity:
(2) $$\min_{w}\;\ell_{k}\left(w\right)+\frac{\mu }{2}{∥w-w_{t}∥}^{2}$$
  where $\ell_{k}\left(w\right)$ is the local loss function and μ is the proximal coefficient. The aggregation performed at the global server follows the same weighted averaging strategy as FedAvg, while the proximal term improves training stability under non‐IID data distributions [33].
- **Federated Normalized Averaging (FedNova)**: FedNova handles the bias introduced by heterogeneous local computation across clients [34]. Let $\Delta_{k}=w_{t}-w_{t}^{\left(k\right)}$ denote the local model update and $\tau_{k}$ the number of local update steps at client k. The global update computed by the global server is:
(3) $$w_{t+1}=w_{t}-\sum_{k=1}^{K}\frac{n_{k}}{n}\frac{\Delta_{k}}{\tau_{k}}$$
- **Federated Stochastic Gradient Descent (FedSGD)**: In FedSGD, each participating client calculates gradients on its local data and sends them directly to the global server without performing multiple local training epochs. The global model is updated by aggregating the client gradients as follows:
(4) $$w_{t+1}=w_{t}-\eta \sum_{k=1}^{K}\frac{n_{k}}{n}\nabla \ell_{k}\left(w_{t}\right)$$
  where η represents the learning rate and $\nabla \ell_{k}\left(w_{t}\right)$ represents the gradient of the local loss function at client k. FedSGD closely resembles centralized stochastic gradient descent and provides stable convergence, although it incurs higher communication overhead compared to multi‐epoch aggregation methods [37].

The aggregation algorithms implemented in the proposed work are summarized in Table 7. To examine how different aggregation strategies influence model learning, these federated aggregation algorithms are implemented. FedAvg serves as a foundational method that combines updates by averaging the local models in a manner proportional to the sizes of clients' datasets. FedProx is built on top of this approach and adds the proximal regularization term that addresses the issue of divergence whenever there is a mismatch in data distributions on the participating clients. FedNova improves upon this method by modifying the updates according to the amount of computation performed in each client, which diminishes data bias induced by the differences between training efforts across clients. Collectively, these methods enable a systematic analysis of how aggregation strategies affect learning performance in federated environments with varying data and system characteristics.

**TABLE 7:** Functional description of aggregation algorithms.

Algorithm	Aggregation strategy	Key characteristics
FedAvg	Weighted averaging of client model updates based on local data size	Simple, efficient; performs well under IID data
FedProx	FedAvg with proximal regularization term $\frac{\mu }{2}{∥w-w_{t}∥}^{2}$	Stabilizes training under non‐IID data; μ=0.01
FedNova	Normalizes local updates through several local training steps	Mitigates client drift; handles heterogeneous workloads
FedSGD	Aggregates gradients computed on local mini‐batches	Communication‐intensive; slower convergence

#### 3.3.6 Proposed Algorithm

The overall training procedure carried out (i.e., client selection, HFL and VFL local training, secure communication, and server‐side aggregation) in the proposed work is described in Algorithm 1.

ALGORITHM 1 **Federated Learning Workflow for Horizontal and Vertical Settings**

**Table:** 

1:	Initialize global model parameters $w_{0}$ at the central server
2:	**for** each communication round t=1,⋯,T **do**
3:	Randomly select a subset of participating clients $S_{t}$
4:	**for** each client $k\in S_{t}$ **in parallel** **do**
5:	**if** Horizontal Federated Learning (HFL) **then**
6:	Receive global model $w_{t}$
7:	Train the local model on private data for E epochs
8:	Compute local model update $\Delta_{k}$
9:	Securely transmit $\Delta_{k}$ to the server
10:	**else if** Vertical Federated Learning (VFL) **then**
11:	Compute local feature embeddings from private inputs
12:	Encrypt and transmit embeddings to the server
13:	Receive aggregated gradients from the server
14:	Update local model parameters accordingly
15:	**end if**
16:	**end for**
17:	**if** HFL **then**
18:	Aggregate client updates using the selected aggregation algorithm
19:	Update the global model to obtain $w_{t+1}$
20:	**else if** VFL **then**
21:	Aggregate encrypted gradients at the server
22:	Update server‐side model parameters
23:	**end if**
24:	**end for**
25:	**return** Final trained federated model

#### 3.3.7 Performance Evaluation Metrics

The performance of the federated learning framework implementation for skin cancer detection was evaluated using the following metrics:

- **Performance Evaluation**: The model's performance is assessed using the various significant parameters such as accuracy, precision, recall, and F1‐score. Sensitivity and specificity are also evaluated to account for rare lesion types. In addition to that, AUC‐ROC is also calculated to measure overall reliability and agreement with true labels.
- **System and Federated Learning Metrics**: The data transfer cost between client nodes and the server node is calculated considering the time required in each global training round. During the evaluation of the federated learning framework, system‐level metrics were considered, including communication rounds, convergence behaviour, and computational requirements, in addition to classification performance.
- **Feature and Reproducibility Analysis**: Feature embeddings are visualized with t‐SNE to evaluate the separability of lesion classes across clients. Also, the variance of local model performance is analyzed to understand the effect of non‐identically distributed data among participating clients. In addition, with different random seeds, experiments are conducted repeatedly to analyze the stability and reproducibility of the proposed framework.

## 4 Results and Discussion

**FIGURE 4:**
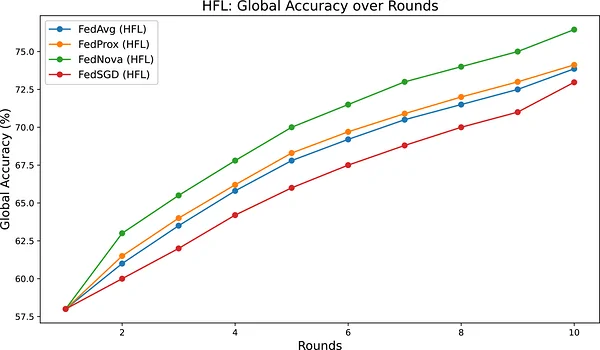
Global accuracy over rounds for HFL model trained on the ISIC dataset.

**FIGURE 5:**
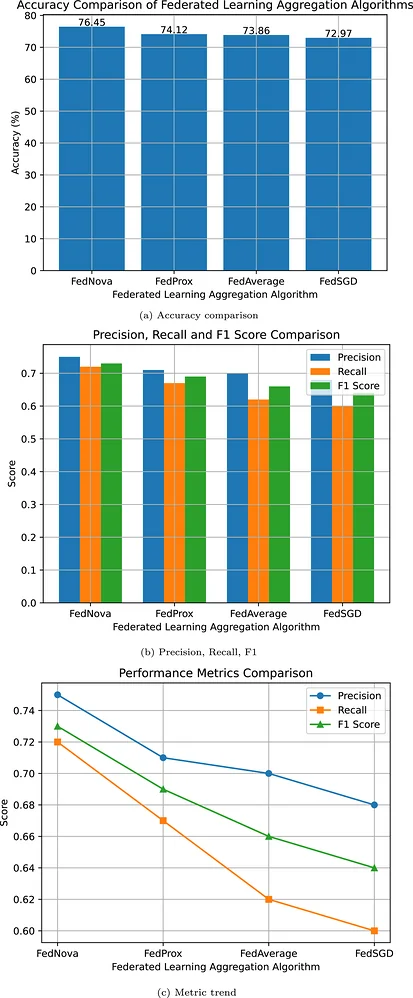
Performance parameter comparisons of aggregation algorithms under HFL settings.

**FIGURE 6:**
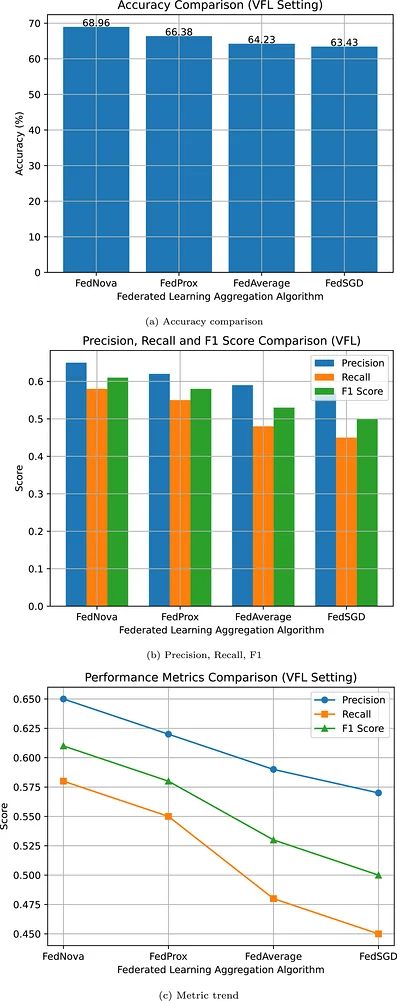
Performance parameter comparisons of aggregation algorithms under VFL settings.

**FIGURE 7:**
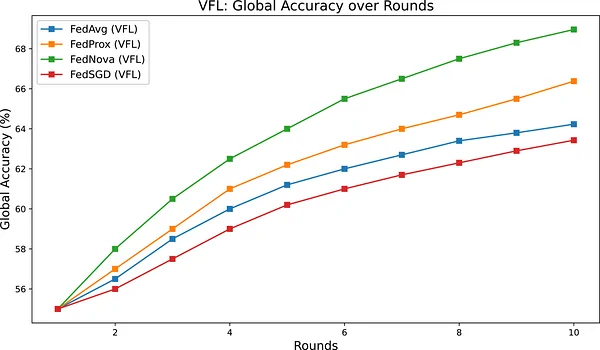
Global accuracy over rounds for VFL model trained on the ISIC dataset.

**FIGURE 8:**
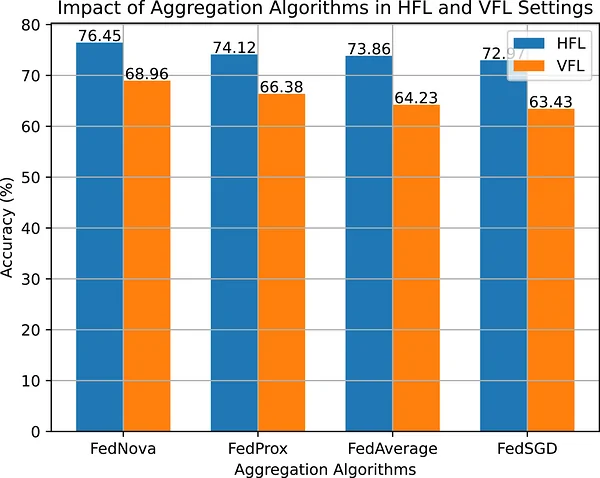
Impact of aggregation algorithms in HFL and VFL settings.

The implementation results show the effect of different aggregation algorithms on skin cancer classification in both HFL and VFL settings. In the HFL settings, FedNova attains the maximum classification accuracy, whereas FedProx and FedAverage results are identical, with lower metric values. This suggests that these methods are moderately sensitive to differences in data distribution among clients. Among all, FedSGD shows the lowest results, due to its high communication requirement and slower convergence. The comparisons of various parameters' results of HFL settings are presented in Figures 4 and 5. While analyzing VFL, compared to HFL settings, there is a measurable fall in performance across all aggregation algorithms. This may be due to the distributed feature space and limited label availability in VFL settings, resulting in complex collaborative model training. Even with these challenges, FedNova relatively outperforms the other aggregation algorithms, which demonstrates its strength in handling client diversity. Figures 6 and 7 further represent the comparison of all parameters and patterns, showing clear differences in accuracy and other parameters that favor FedNova. Overall, the results state that the selection of the aggregation algorithm and the data distribution notably affect the model's final performance. Performance comparisons of aggregation algorithms in HFL and VFL settings are shown in Figure 8. The implementation results (refer to Table 8 and Figure 8, respectively) demonstrate that the FedNova outperforms in all evaluation parameters, viz., accuracy, precision, recall, and F1‐score.

**TABLE 8:** Performance comparison of centralized ResNet50 baseline and aggregation algorithms under HFL and VFL settings.

Setting	Algorithm	Accuracy	Precision (macro)	Recall (macro)	F1‐score (macro)
Centralized	ResNet50	66.07 ± 0.35%	0.512 ± 0.027	0.308 ± 0.003	0.315 ± 0.007
HFL	FedNova	76.45%	0.75	0.72	0.73
	FedProx	74.12%	0.71	0.67	0.69
	FedAverage	73.86%	0.70	0.62	0.66
	FedSGD	72.97%	0.68	0.60	0.64
VFL	FedNova	68.96%	0.65	0.58	0.61
	FedProx	66.38%	0.62	0.55	0.58
	FedAverage	64.23%	0.59	0.48	0.53
	FedSGD	63.43%	0.57	0.45	0.50

To present a fair comparison, the centralized ResNet50 baseline was evaluated with the same preprocessing pipeline, train/validation/test split, and evaluation process as the federated learning experiments. The results shown in Table 8 reflect that the centralized baseline results showed an average test accuracy of 66.07 ± 0.35%. Out of all the federated learning methods, HFL with the FedNova aggregation algorithm exhibited the best classification accuracy of 76.45%, while VFL also provided satisfactory classification performance. This shows that the federated learning framework retains its capability for efficient classification while ensuring data privacy.

### 4.1 Model Performance

The performance of the models is evaluated using significant parameters such as accuracy, precision, recall, and F1‐score. It is noticed that FedNova in the HFL setting achieved the highest accuracy (76.45%) and performed better compared to other evaluated aggregation algorithms over all reported performance metrics.

**FIGURE 9:**
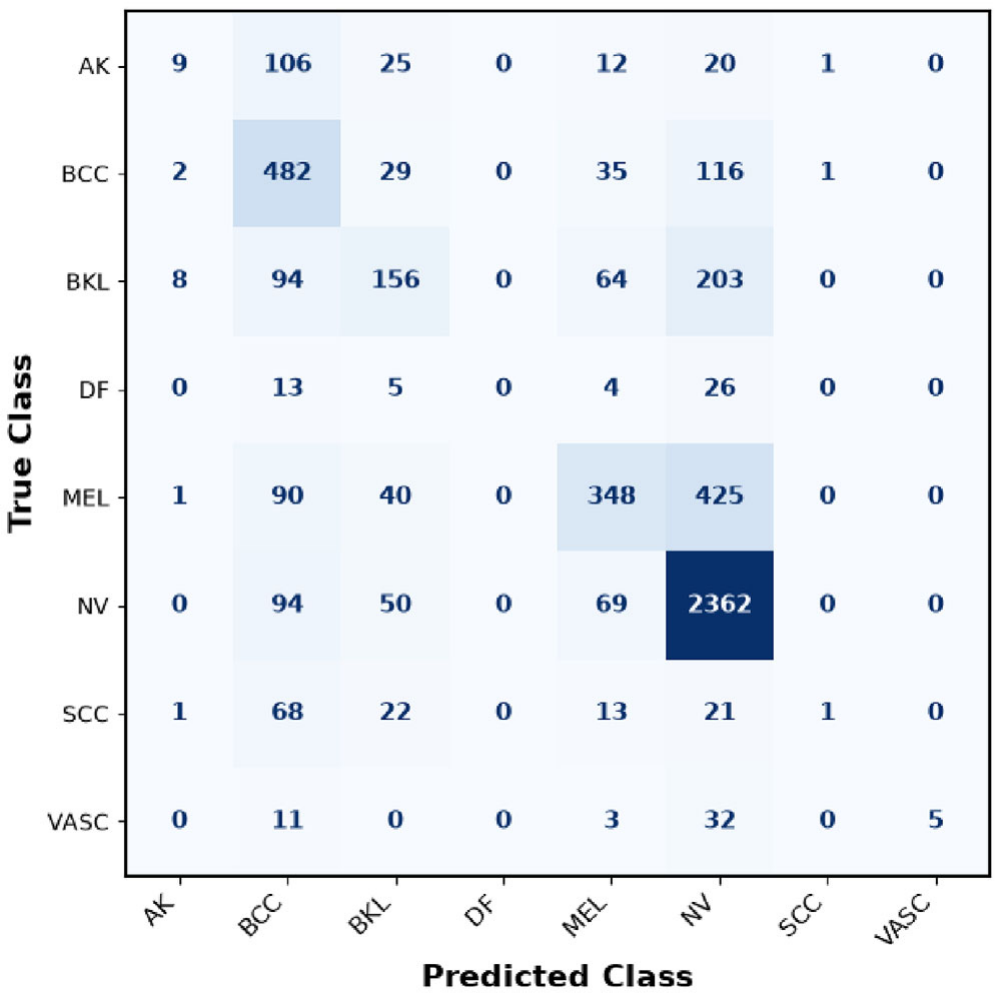
Confusion matrix of the centralized ResNet50 model on the ISIC 2019 test dataset.

In addition to the evaluation metrics, an in‐depth class‐wise analysis was conducted, applying the concepts and metrics of precision, sensitivity (recall), specificity, and F1‐score for each of the lesions present. The confusion matrix showing the results for the centralized baseline is presented in Figure 9, and the class‐wise results are mathematically represented through Table 9. The results show that the NV class had the highest recall and F1 score due to the fact that this class has a higher representation in the data. In contrast, although some of the minority lesion categories such as AK, DF, SCC, and VASC had high specificity scores, they showed low sensitivity, which underlines the negative influence of severe class imbalance. It is also observed that MEL among the malignant lesions showed an average performance of classification, which means that melanoma detection is still a challenging task. Based on these findings, it can be concluded that the proposed framework shows good results; however, it requires further work concerning underrepresented classes.

**TABLE 9:** Class‐wise performance evaluation of the centralized ResNet50 baseline.

Class	Precision	Sensitivity (recall)	Specificity	F1‐score	Support
AK	0.4286	0.0520	0.9975	0.0928	173
BCC	0.5031	0.7248	0.8919	0.5940	665
BKL	0.4771	0.2971	0.9624	0.3662	525
DF	0.0000	0.0000	1.0000	0.0000	48
MEL	0.6350	0.3850	0.9520	0.4793	904
NV	0.7370	0.9173	0.6617	0.8173	2575
SCC	0.3333	0.0079	0.9996	0.0155	126
VASC	1.0000	0.0980	1.0000	0.1786	51

*Note*: Overall ROC–AUC of the centralized ResNet50 baseline was 0.8755 (Macro AUC) and 0.9353 (Micro AUC).

### 4.2 System and Training Observations

The communication cost and computation time per global round are recorded for all algorithms. The results in Figures 4 and 7 demonstrate that FedNova achieves the fastest improvement in global accuracy during the first rounds of communication in both the HFL and VFL scenarios. The curves indicate that by the 10th round of communication, FedNova achieved about 76.5% accuracy in HFL and 69% accuracy in VFL. FedProx achieved approximately 74% and 66.4% accuracy, while FedAvg achieved approximately 73.5% and 64.2% accuracy, respectively. The slowest results throughout the rounds belong to FedSGD, which achieved about 72.8% accuracy in HFL and 63.5% in VFL after the 10th round of communication. This shows that FedNova demonstrates the highest rate of convergence and the best classification results compared to other aggregation methods.

### 4.3 Feature Analysis

**FIGURE 10:**
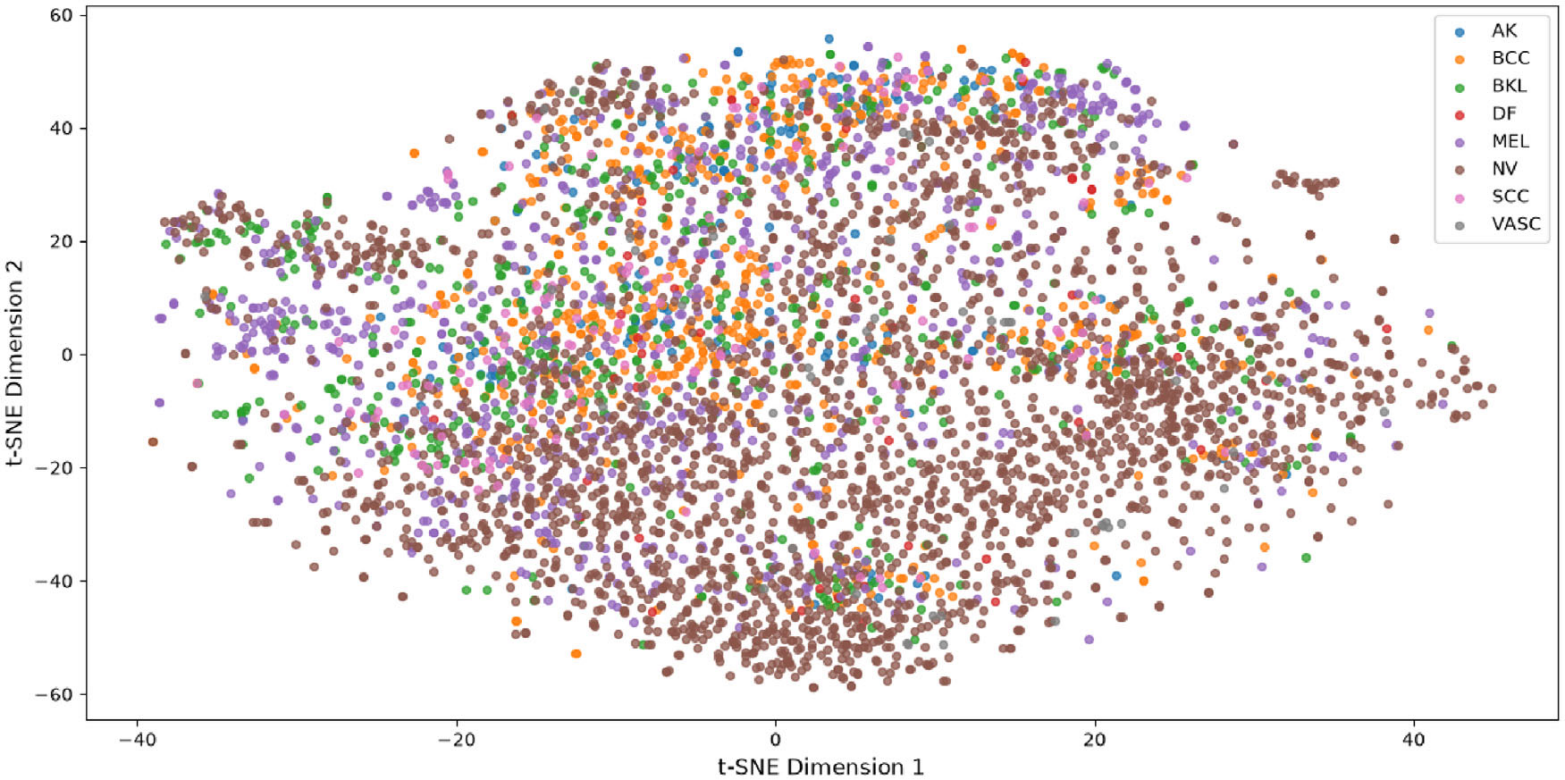
t‐SNE visualization of the learned feature space.

t‐SNE is used to visualize feature embeddings. It presents consistent separation of lesion classes across multiple participating clients. The variance in local model performance is analyzed to assess the impact of non‐IID data. It reflects that FedNova and FedProx mitigate divergence more effectively compared to FedAvg or FedSGD. The t‐SNE visualization of the features provided by the centralized ResNet50 model is portrayed in Figure 10. The visualization shows that the most prominent lesion type (NV) occupies rather closed areas of the feature space, while a number of less represented lesion types overlap significantly in the latent space. This observation is consistent with the results demonstrated in Table 9, where the minor classes yielded much lower sensitivity and F1 scores due to the severe imbalance among classes. Overall, the feature representations learned by the mentioned model show a reasonable level of separability among the different prominent classes, but are still faced with challenges posed by lesser‐represented classes.

#### 4.3.1 Reproducibility Analysis

In order to verify the reproducibility of the centralized baseline, the model was trained three times independently with three different random seeds (42, 52, and 62), while keeping all preprocessing, dataset splitting, and evaluation processes identical. The individual results of the experiments are given in Tables 10 and 11. The mean test accuracy of the centralized baseline was 66.07 ± 0.35% (95% confidence interval: 65.19%–66.95%). The macro precision, recall, and F1‐score also showed small differences between runs, indicating that the implemented training pipeline is consistently reproducible across the same set of experiments.

**TABLE 10:** Repeated experimental results of the centralized ResNet50 baseline.

Random seed	Accuracy (%)	Precision	Recall	F1‐score
42	66.37	0.5143	0.3103	0.3180
52	66.15	0.5378	0.3089	0.3205
62	65.68	0.4849	0.3040	0.3073

**TABLE 11:** Statistical summary of the repeated centralized experiments.

Performance metric	Mean ± standard deviation	95% Confidence interval
Accuracy (%)	66.07 ± 0.35	65.19–66.95
Precision	0.512 ± 0.027	0.446–0.578
Recall	0.308 ± 0.003	0.300–0.316
F1‐score	0.315 ± 0.007	0.298–0.333

### 4.4 Framework Challenges

Several challenges are identified during implementation. Non‐IID data among participating clients resulting divergence in local models and affects global convergence. Class imbalance for rare lesion images remains a major concern. Communication overhead and client resource limitations are the main practical constraints.

The comparative analysis presented illustrates the impact of different federated learning paradigms and aggregation schemes on the classification of skin cancers while preserving patient privacy in a common experimental setup. Through comparing the HFL and VFL using identical preprocessing, model architecture and evaluation criteria, we are able to clarify how aggregation scheme differences impact classification performance. Thus, these results provide practical advice for deciding among different federated learning configurations when analysing distributed medical imagery and maintaining patient confidentiality.

## 5 Conclusion

A proposed federated deep learning framework for detecting skin cancer obtained a higher performance without compromising patient privacy. Four aggregation approaches, viz. FedAvg, FedNova, FedSGD, and FedProx in vertical and horizontal FL settings are implemented and observed to analyze their impact on results. Experimental results demonstrated that HFL outperformed VFL in terms of accuracy for classifying skin cancer. FedNova was identified as the best‐performing aggregation algorithm compared to other evaluated aggregation functions, with its maximum performance values (i.e., Accuracy = 76.45%, Macro Precision = 0.75, Macro Recall = 0.72, and Macro F1 = 0.73) achieved in the HFL setting compared to the VFL implementation, which achieved an overall accuracy of 68.96%.

Future work will cover the analysis and impact of the lesion segmentation process on federated skin cancer classification performance and examine strategies of multimodal learning for further enhancing diagnostic accuracy.

## Author Contributions

**Bela Shrimali**: conceptualization, methodology, validation, writing – original draft. **Mohit Ginglani and Rushi Shah**: conceptualization, software, validation, formal analysis, visualization, investigation, writing – original draft preparation. **Rebakah Geddam**: methodology, formal analysis, writing – review & editing. **Hemant Ghayvat**: conceptualization, supervision, methodology – offered continuous advisory support.

## Funding

The authors have nothing to report.

## Consent

All participants provided informed consent for the publication of the results.

## Conflicts of Interest

All authors agreed with the content, and all gave explicit consent to submit, and that they obtained consent from the responsible authorities at the institute/organization where the work has been carried out before the work is submitted.

## Data Availability

The data that support the findings of this study are available in ISIC 2019 Skin Lesion images and for classification at https://www.kaggle.com/datasets/salviohexia/isic‐2019‐skin‐lesion‐images‐for‐classification. These data were derived from the following resources available in the public domain. ISIC 2019: https://www.kaggle.com/datasets/salviohexia/isic‐2019‐skin‐lesion‐images‐for‐classification; HAM10000: https://www.kaggle.com/datasets/kmader/skin‐cancer‐mnist‐ham10000. The underlying code for this work is currently maintained in a private GitHub repository as it is under the peer‐review process. After the acceptance of the manuscript, it will be made publicly available at: https://github.com/BS253183/SkinCancer_FL.
